# Supplementary material for: BRCA mutational status shapes the stromal microenvironment of pancreatic cancer linking clusterin expression in cancer associated fibroblasts with HSF1 signaling
Source: Nat Commun. 2022 Oct 31;13:6513. doi: 10.1038/s41467-022-34081-3 (PMC9622893; doi:10.1038/s41467-022-34081-3)
Supplement: Supplementary file 1 — Supplementary Information [file 41467_2022_34081_MOESM1_ESM.pdf]

BRCA mutational status shapes the stromal microenvironment of pancreatic cancer linking clusterin expression in cancer associated fibroblasts with *HSF1* signaling

Supplementary Figures 1-6

Shaashua et al; Supplementary Figure 1

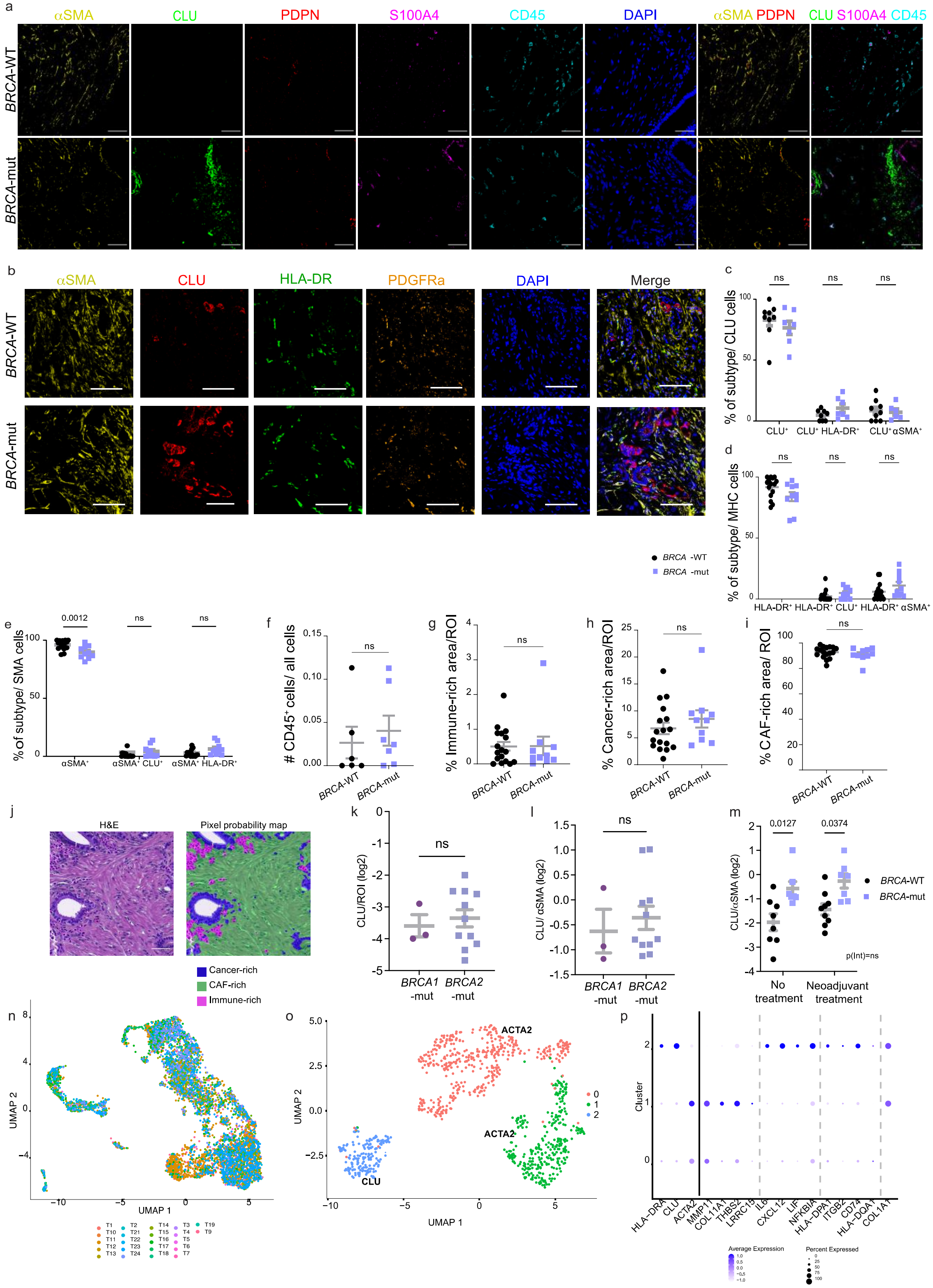

Supplementary Figure 1. PDAC stroma is comprised of three distinct CAF subtypes. (a-b) FFPE tumor sections from 5 *BRCA*-WT and 3 *BRCA*-mut PDAC patients were stained by MxIF for PDPN, PDGFRA, S100A4, CLU, αSMA, CD45 and cytokeratin (CK). DAPI was used to stain nuclei. Scale bar is 50μm for a and 100μm for b. Panel a was repeated twice, and panel b was done once; for both panels n=5 *BRCA*-WT and n=3 *BRCA*-mut PDAC patients. (c-e) MxIF was performed on FFPE tumor sections from 7 *BRCA*-mut and 6 *BRCA*-WT PDAC patients using antibodies for αSMA, CLU, HLA-DR, CK, and CD45. DAPI was used to stain nuclei. The fraction of each subtype out of all the cells that expresses CLU (c), HLA-DR (d), and αSMA (e) is presented. Data was analyzed using two way ANOVA and are presented as mean ± SEM (f) The relative abundance of $CD45^+$ cells was calculated, averaged for each patient sample, and is presented as mean ± SEM across patients. Data were analyzed using unpaired t-test (g-j) H&E stained FFPE tumor sections from 17 *BRCA*-WT and 10 *BRCA*-mut patients were scanned and analyzed employing pixel classification using QuPath software. The percent of immune-rich (g), cancer-rich (h), and CAF-rich (i) areas out of an ROI of the tumor area (excluding normal adjacent tissue) was calculated. Representative H&E images before (left) and after (right) pixel classification are shown in (j). Blue marks cancer-rich areas, green marks CAF-rich areas, and pink marks immune-rich. Scale bar - 50 μm. Data were analyzed using unpaired t-test and are presented as mean ± SEM (k-m) FFPE tumor sections from 17 *BRCA*-WT and 14 *BRCA*-mut carriers (n=11 for *BRCA2* and n=3 for *BRCA1*) PDAC patients were stained by MxIF for CLU, αSMA, and cytokeratin (CK). DAPI was used to stain nuclei. Images were analyzed using ImageJ software, CK^- regions were defined as regions of interest (ROIs) and the area stained by each CAF marker was calculated, divided by the ROI and averaged for each patient sample. Quantification of the CLU^+ area (k) and the ratio between CLU^+ and αSMA^+ CAFs (l) is presented for tumor samples from *BRCA1 vs*. *BRCA2* mutation carriers. Quantification of the ratio between CLU^+ and αSMA^+ CAFs for tumor samples from neoadjuvant-treated *vs.* non-treated patients is presented (m). Data are presented as mean ± SEM. ns marks p-values greater than 0.05. Data in k-l were analyzed using unpaired t-test. Data in m were analyzed using two way ANOVA test (n) UMAP dot plot of 6,405 cells defined as fibroblast and stellate cells in the dataset from Peng et al, color-coded by patient number. (o) UMAP dot plot of 954 cells based on scRNA-seq of human fibroblasts from [12], color-coded for the indicated cell clusters defined by a local moving clustering algorithm. The clusters that differentially express *ACTA2 and CLU* are indicated. (p) Dot plot visualization of gene expression of the indicated CAF markers. Source data are provided as a Source Data file.

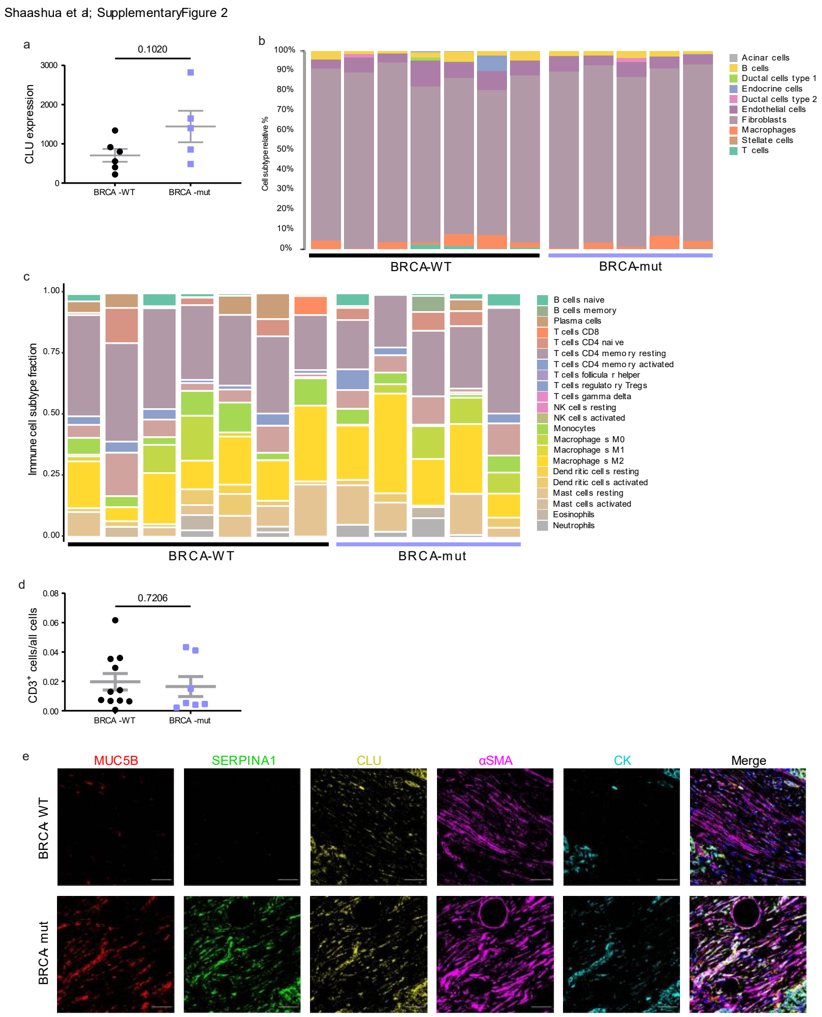

Supplementary Figure 2. Characterization of CAF-rich regions from *BRCA*-WT and *BRCA*-mut PDAC. RNA-seq data from laser capture microdissected CAF-rich regions of 7 *BRCA*-WT and 5 *BRCA*-mut PDAC patients was analyzed. (a) *CLU* expression levels were assessed in *BRCA*-mut (n=5) and *BRCA*-WT (n=6) tumors. Data are presented as mean ± SEM using unpaired t-test (b) CIBERSORT analysis was used to estimate the relative abundance of individual cell types in the samples collected by LCM from 7 *BRCA*-WT and 5 *BRCA*-mut PDAC patients (c) CIBERSORT analysis from 7 *BRCA*-WT and 5 *BRCA*-mut PDAC patients was used to estimate immune cell composition of *BRCA*-mut and *BRCA*-WT CAF-rich regions. The predicted fraction of each immune cell population is presented. (d) 7 *BRCA*-mut and 11 *BRCA*-WT tumors were stained for CD3 by MxIF. The relative number of $CD3^+$ cells was calculated and is presented as mean ± SEM using unpaired t-test. (e) FFPE tumor sections from 9 *BRCA*-mut and 9 *BRCA*-WT PDAC patients were stained by MxIF using antibodies for the indicated proteins. DAPI was used to stain nuclei. Scale bar, 50μm. Representative images are presented. Source data are provided as a Source Data file.

Shaashua et al; Supplementary Figure 3

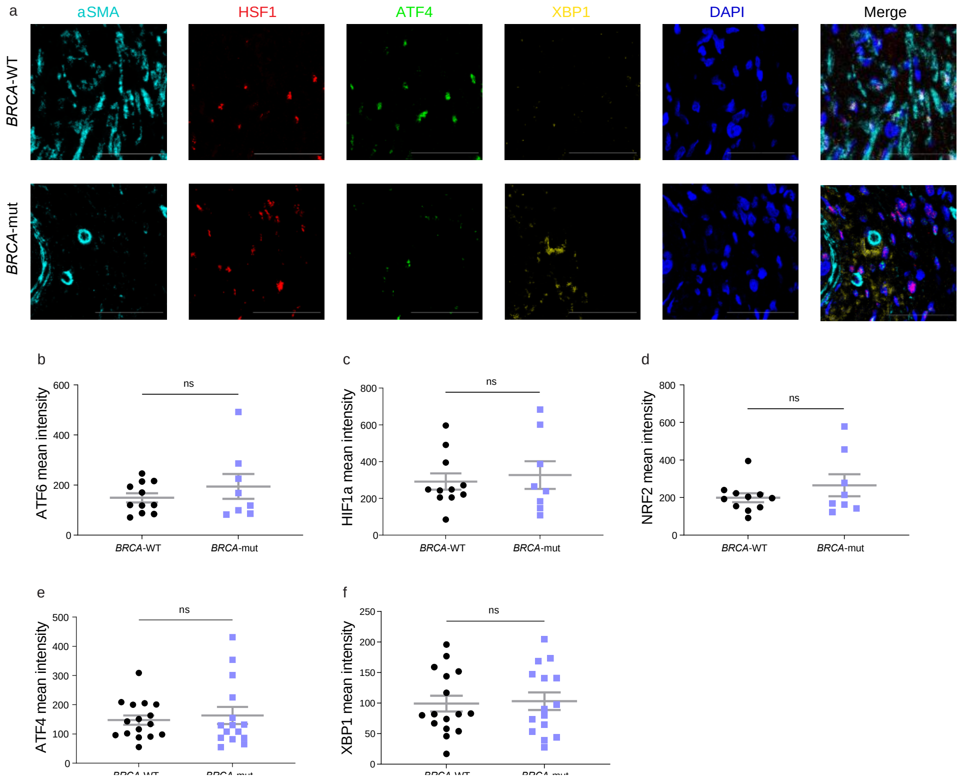

Supplementary Figure 3. CAFs from *BRCA*-WT and *BRCA*-mut PDAC exhibit similar activation levels of the responses to ER stress, integrated stress, hypoxia, and oxidative stress. FFPE tumor sections from *BRCA*-mut and *BRCA*-WT PDAC patients were stained by MxIF using antibodies for the indicated proteins. (a) Representative images of insets (From images shown in Figure 3B) are shown. DAPI was used to stain nuclei. Scale bar, 50µm. (b-f) 3-5 images per patient were analyzed using ImageJ software to quantification the nuclear staining (mean intensity) of ATF6 (b), HIF1a (c), NRF2 (d), ATF4 (e), and XBP1 (f) within all stromal cells in *BRCA*-mut (n=8 for b-d; n=15 for e-f) and *BRCA*-WT samples (n=11 for b-d; n=16 for e-f). Data are presented as mean across patients ± SEM using unpaired t-test. ns marks p-values greater than 0.05. Source data are provided as a Source Data file.

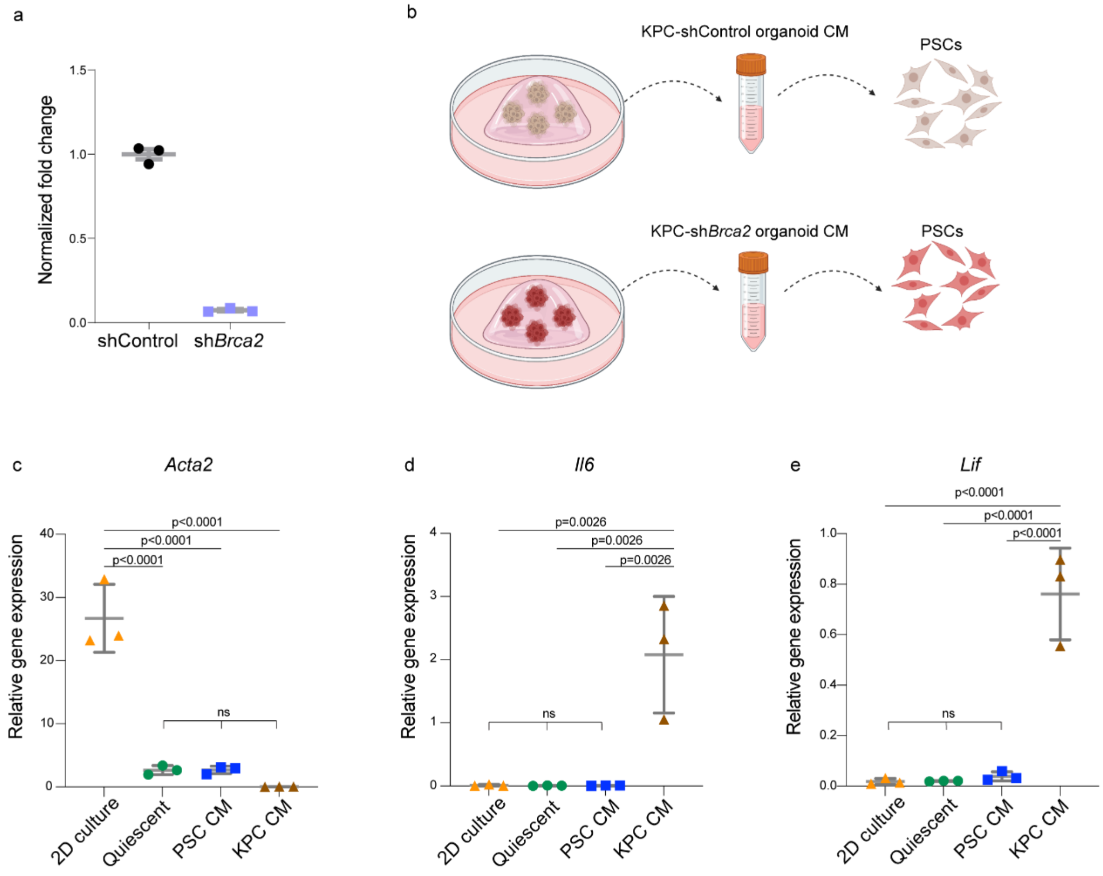

Supplementary Figure 4. PSCs grown in 3D with cancer conditioned medium downregulate myofibroblast-related genes and induce inflammatory genes. (a) KPC organoids were transfected with sh*Brca*2 or shControl. *Brca*2 levels were measured by qRT-PCR compared to non-targeting shControl. Data are presented as mean ± SEM. The analysis was conducted for each experiment involving sh*Brca2*; A representative analysis of 3 technical replicates is shown, two additional representative repeats are displayed in the source data file. (b) Schematic representation of the workflow. Immortalized PSCs were treated with CM collected from either KPC-shControl or KPC-sh*Brca2* organoids grown in 3D. PSCs were seeded in Matrigel for 4 days in growth medium (DMEM with 10% FBS). CM from KPC-shControl or KPC-sh*Brca2* organoids was added for 4 additional days, or cells were left in growth medium, as control. Then, the PSCs were isolated from the Matrigel dome, RNA was extracted and RNA-seq was performed. The image was generated using biorender.com (c-e) Immortalized PSCs were grown in 2D culture or in Matrigel for 4 days in growth medium after which they were either isolated from the 2D culture or Matrigel (quiescent) or treated in Matrigel with CM from PSCs or from KPC organoids for 4 additional days. The expression levels of *Acta2* (c), *Il6* (d) and *Lif* (e), were measured by qRT-PCR. One-way ANOVA was performed on normalized values (a), or raw values (c-e). Tukey's test was performed to adjust for multiple comparisons. n=3 biologically independent wells. Source data are provided as a Source Data file.

Shaashua et al; Supplementary Figure 5

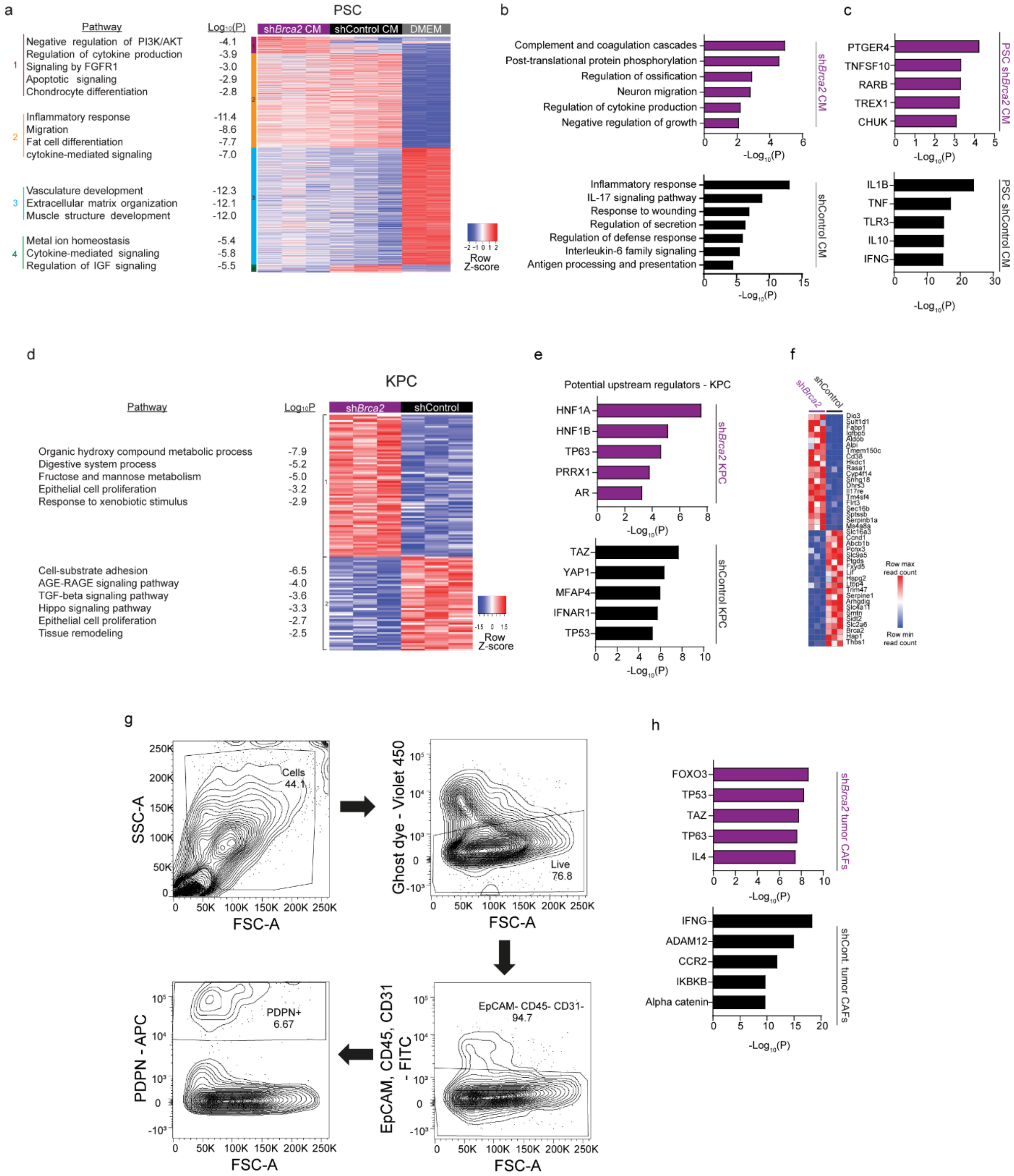

Supplementary Figure 5. Loss of *Brca2* leads to transcriptional rewiring of KPC cells and affects the transcriptional profile of PSCs. (a-c) RNA-seq analysis of PSCs conditioned by KPC-organoid cultures. (a) A heatmap representing hierarchical clustering of the DE genes between the three treatments (right), and pathways enriched in each of the designated clusters with their corresponding p-values (left). n=2 biologically independent domes for DMEM treated PSCs and n=3 biologically independent domes for shControl-CM-treated and KPC-shBrca2-CM-treated PSCs. Pathway analysis was performed using Metascape, selected pathways are shown, see Supplementary Table 8 (tabs 2-5) for the full lists. (b-c) Pathways for (a), enriched in KPC-sh*Brca2*-CM-treated PSCs (compared to shControl) and in KPC-shControl-CM-treated PSCs (compared to sh*Brca2*). Pathway analysis was performed using Metascape, selected pathways are shown, see Supplementary Table 8 (tabs 8-9) for the full list. (d-f) Analysis of bulk RNA-seq from KPC-sh*Brca2* (n=3 mice) and KPC-shControl cells (n=3 mice). The cells were cultured (2D cultures) for two days, lysed, and RNA was extracted and sequenced. (d) A heatmap representing hierarchical clustering of DE genes between KPC-sh*Brca2* and KPC-shControl cells (right), and the pathways enriched in each cluster (left). Pathway analysis was performed using Metascape, selected pathways are shown, see Supplementary Table 9 for the full lists. (e) Potential upstream regulators of the DE genes between KPC-sh*Brca2* and KPC-shControl cells were predicted using IPA (see Methods). The five most significant regulators and their corresponding p-values are presented, see Supplementary Table 9 for the full lists. (f) A heatmap representing the 20 most upregulated genes in KPC-sh*Brca2*, and 20 most upregulated genes in KPC-shControl cells. (g) Gating strategy for sorting CAFs from KPC-sh*Brca2* and KPC-shControl tumors. Representative FACS plots are shown, n=3 mice for KPC-shControl tumors and n=4 mice for KPC-sh*Brca2* tumors (h) IPA prediction of potential upstream regulators in CAFs from KPC-sh*Brca2* (n=4) *vs* KPC-shControl (n=3) tumors. The five most significant regulators and their corresponding p-values are presented, see Supplementary Table 10 for the full list. Source data are provided as a Source Data file.

Shaashua et al; Supplementary Figure 6

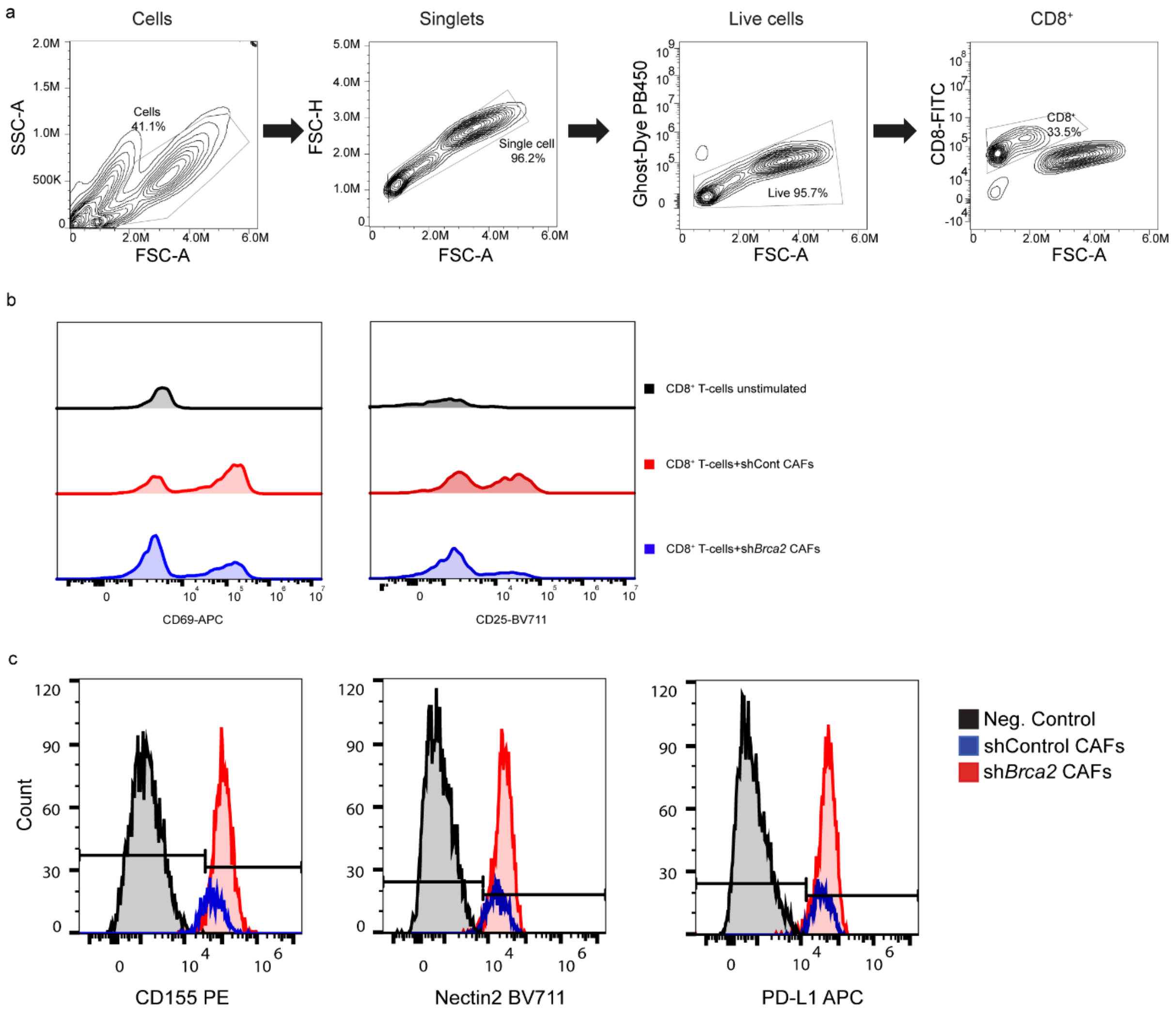

Supplementary Figure 6. Knockdown of *Brca2* in KPC cells promotes an immune regulatory CAF phenotype. (a) Gating strategy for selection of $CD8^+$ T-cells following co-culture with CAFs isolated from primary murine tumors. (b) Representative FACS plot of $CD8^+$ T-cells co-cultured with CAFs from either shControl or sh*Brca2* tumors. T-cells were left either unstimulated or stimulated with CD3/CD28 Dynabeads, and stained for CD69 and CD25 to assess activation via FACS. (c) Representative FACS plot of primary CAFs isolated from shControl and sh*Brca2* tumors and stained for the inhibitory checkpoint ligands CD155, Nectin2, and PD-L1. Source data are provided as a Source Data file.
