# Supplementary material for: BRCA mutational status shapes the stromal microenvironment of pancreatic cancer linking clusterin expression in cancer associated fibroblasts with HSF1 signaling
Source: Nat Commun. 2022 Oct 31;13:6513. doi: 10.1038/s41467-022-34081-3 (PMC9622893; doi:10.1038/s41467-022-34081-3)
Supplement: Supplementary file 3 — Description of Additional Supplementary Files [file 41467_2022_34081_MOESM3_ESM.pdf]

Title: Supplementary Data 1
Description: Clinical data_Cohort 1

Title: Supplementary Data 2
Description: Single cell analysis_Peng dataset

Title: Supplementary Data 3
Description: Single cell analysis_Elyada dataset

Title: Supplementary Data 4
Description: RNA seq_LCM patient samples

Title: Supplementary Data 5
Description: CIBERSORT analyses

Title: Supplementary Data 6
Description: Clinical data_Cohort 2

Title: Supplementary Data 7
Description: MassSpec_CM

Title: Supplementary Data 8
Description: PSC_RNA-seq

Title: Supplementary Data 9
Description: KPC_RNA-seq

Title: Supplementary Data 10
Description: CAF_RNA-seq

Title: Supplementary Data 11
Description: HSF1 targets

Title: Supplementary Data 12
Description: Abs

Title: Supplementary Data 13
Description: Primer sequences
